# Preparing pathological data to develop an artificial intelligence model in the nonclinical study

**DOI:** 10.1038/s41598-023-30944-x

**Published:** 2023-03-08

**Authors:** Ji-Hee Hwang, Minyoung Lim, Gyeongjin Han, Heejin Park, Yong-Bum Kim, Jinseok Park, Sang-Yeop Jun, Jaeku Lee, Jae-Woo Cho

**Affiliations:** 1 Toxicologic Pathology Research Group, Department of Advanced Toxicology Research, Korea Institute of Toxicology, Daejeon, 34114 Korea; 2 Department of Advanced Toxicology Research, Korea Institute of Toxicology, Daejeon, 34114 Korea; 3 Research and Development Team, LAC Inc., Seoul, 07807 Korea

**Keywords:** Computational models, Toxicology, Pathology

## Abstract

Artificial intelligence (AI)-based analysis has recently been adopted in the examination of histological slides via the digitization of glass slides using a digital scanner. In this study, we examined the effect of varying the staining color tone and magnification level of a dataset on the result of AI model prediction in hematoxylin and eosin stained whole slide images (WSIs). The WSIs of liver tissues with fibrosis were used as an example, and three different datasets (N20, B20, and B10) were prepared with different color tones and magnifications. Using these datasets, we built five models trained Mask R-CNN algorithm by a single or mixed dataset of N20, B20, and B10. We evaluated their model performance using the test dataset of three datasets. It was found that the models that were trained with mixed datasets (models B20/N20 and B10/B20), which consist of different color tones or magnifications, performed better than the single dataset trained models. Consequently, superior performance of the mixed models was obtained from the actual prediction results of the test images. We suggest that training the algorithm with various staining color tones and multi-scaled image datasets would be more optimized for consistent remarkable performance in predicting pathological lesions of interest.

## Introduction

Histopathological images include influential information referring to the cell anatomy and tissues of organisms, which can be crucial for the final decision procedure of effective therapeutics for diseases such as cancer^1,2^. Traditional pathological diagnosis is performed by observing the stained specimen on a glass slide using a microscope^3^. The development of whole-slide scanners has allowed for the digitization of histopathological images by generating whole-slide images (WSI), which have facilitated the pathologist's workflow through digital pathology^4^. In addition, a large number of WSIs can be accumulated, which accelerates the adaptation of digital image analysis methods to aid in pathology-related tasks, including diagnosis^5^.

After the dissemination of WSIs, digital approaches to histopathological image analysis in digital pathology have focused primarily on low-level image analysis tasks, such as staining normalization, nuclear segmentation, and feature extraction, followed by the construction of classification models using classical machine learning methods^4^. As a result of low-level image analysis, computer-aided diagnosis (CAD) using histopathological images has become a standard clinical diagnostic procedure for cancer detection, and it is now one of the major stages in the histopathological imaging and diagnosis process^6^. The first stage of the diagnosis process is categorizing a WSI or multiple WSIs for a disease, which is essential for supervised learning tasks. The classification accuracy of the machine learning system is different from that of a human pathologist^7^; therefore, it can be improved using CAD and could prevent oversight by investigating all pixels within WSIs^5^. After categorizing the WSIs, the other diagnosis tasks are the detection or segmentation of regions of interest (ROI) such as the tumor region in WSI^8^, scoring of immunostaining^9,10^, cancer staging^7,11,12^, mitosis detection^7,13^, gland segmentation^14,15^, and detection and quantification of vascular invasion^16^. These are the labeling stages of AI algorithm training. The performance of the supervised learning AI model is greatly affected by data preparation for training and testing, which could be the key to overcoming the obstacles to applying AI in pathological diagnosis^17^.

There are various obstacles to overcome in preparation for training an AI algorithm with WSI of organ tissue, such as the large size of the image and insufficiently labeled images. Numerous researchers have attempted to solve these problems by increasing the efficiency of label data, utilizing weak labels or unlabeled information, or utilizing models/parameters for other tasks. However, the magnification and staining variation of the image are also important issues in training an AI algorithm for implementation in an automated diagnosis. Histopathology images were captured in several stages, such as specimen slicing, hematoxylin and eosin staining (H&E), and scanning. At each unwanted anomaly, differences that are unassociated with the underlying biological factors according to the previously mentioned stages and even by different vendors of the scanner could be considered^4,18^. After scanning, a proper level of magnification of the slide images is important. Regardless of the variation in WSI, pathologists acquire different types of information from the cellular to tissue level for pathological diagnosis by changing the magnification of the microscope. To automatically predict the pathological outcome from the tissue slides through the AI algorithm, high-magnification objective images that are more deterministic and informative in the cell base and low-magnification images, which are more optimized for structural information such as glandular, are needed. To achieve this goal, AI researchers have employed image datasets with different levels of magnification^19,20^, conversion to grayscale^21,22^, color normalization^23,24^, and augmentation^25^ in clinical studies to diagnose various cancers. However, the adaptation of an AI algorithm for toxicological pathologic diagnosis in non-clinical studies started later than in clinical studies. Indeed, toxicologic pathological studies have unique challenges when compared to clinical pathology, in terms of the number of slides per the study and differentiation of the normal background lesions from abnormal lesions induced by test items. Recently, many AI-assisted analyses in non-clinical research and toxicologic pathology have been published, focusing on computer-assisted QC, research-driven computational image analysis, computer-assisted abnormality detection, and content-based image retrieval^26^. However, intensive discussion, including experimental proof of preparing a proper dataset for the implementation of an AI algorithm for auto diagnosis, is scarce.

In this study, to discuss the effective application of AI algorithm for toxicologic pathology using in-house H&E stained slides, we trained the Mask R-CNN algorithm with different datasets and evaluated the trained models’ performance. We prepared four datasets with varying staining color tones and magnification and trained the Mask R-CNN algorithm, which predicts an object based on the region and pixel level. The algorithm was trained with a single or mixed dataset, and the trained models’ performance was evaluated using large-scale images than training images to represent their performance on the basis of real-world data. The performance of each model was assessed using three different parameters, precision, recall and accuracy. By doing so, we expect to emphasize that proper dataset composition is important to obtain a reasonable performance by an AI model for detecting lesions of interest.

## Materials and methods

### Data preparation

To investigate the effectiveness of the trained AI model in predicting a pathological lesion in non-clinical pathology, we prepared four datasets with different magnifications and staining tones. One thousand image tiles of 448 × 448 pixels were prepared for each dataset, and pathological lesions and hepatic fibrosis were annotated using the VGG annotator 2.0.1.0 (visual Geometry Group, Oxford University, United Kingdom). An accredited toxicologic pathologist confirmed all the annotations before algorithm training. Whole slide images (WSIs) used for the dataset were obtained from two retrospective studies, N and B, which induced hepatic fibrosis in Sprague–Dawley (SD) rats by N-nitrosodimethylamine (NDMA)^27^. All procedures, inducing hepatic fibrosis in SD rats in studies N and B, were the same, but with different H&E staining conditions. The difference in staining tone between the two studies was identified by analyzing the RGB values using ImageJ (NIH). Representative images of each study are shown in Supplementary Figs. S1 and S2. In addition, 100 images, which had similar staining tones to study B, were prepared from the sectioning slides of study N. A total of 2100 image tiles were obtained from 104 WSIs, the lesions identified on these images were labeled, and total annotations were obtained. The annotated image tiles were split into training, validation, and test datasets using the train_test split function embedded in the scikit-learn package (ratio 7:2:1). Data augmentation was conducted eight times to improve the training dataset using image-augmenting techniques (reverse, rotation, and brightness). The details of the total number of images and annotations used for each dataset can be seen in Table 1.

**Table 1:** The number of images and annotations used for training, validation, and testing according to the dataset.

Number of images or annotations								
	B10 dataset		B20 dataset		N20 dataset		Recut N20 dataset	
	Images	Annotations	Images	Annotations	Images	Annotations	Images	Annotations
Train	701 (5608)	894 (7152)	700 (5600)	763 (6104)	700 (5600)	755 (6040)	–	–
Validation	199	283	200	217	200	212	–	–
Test	100	128	100	109	100	105	100	100
Total	1000 (5907)	1305 (7563)	1000 (5900)	1089 (6430)	1000 (5900)	1072 (6357)	100	100

The numbers in parentheses are augmented numbers of each image for annotations.

### Training of the algorithm and metrics for model performance

#### Model training

All procedures related to algorithm training were performed using an open-source framework for machine learning (Tensorflow 2.1.0, Keras 2.4.3 backend, and PyTorch) powered by an NVIDIA RTX 3090 24G GPU. Torchvision^28^ was applied in the use of the algorithm (its requirements were met in this study). The hyperparameters tuned for network learning were adjusted according to a previous study^27^. All the models were trained with the same hyperparameters, and details can be found Supplementary Fig. S3.

#### Building test model

**Figure 1:**
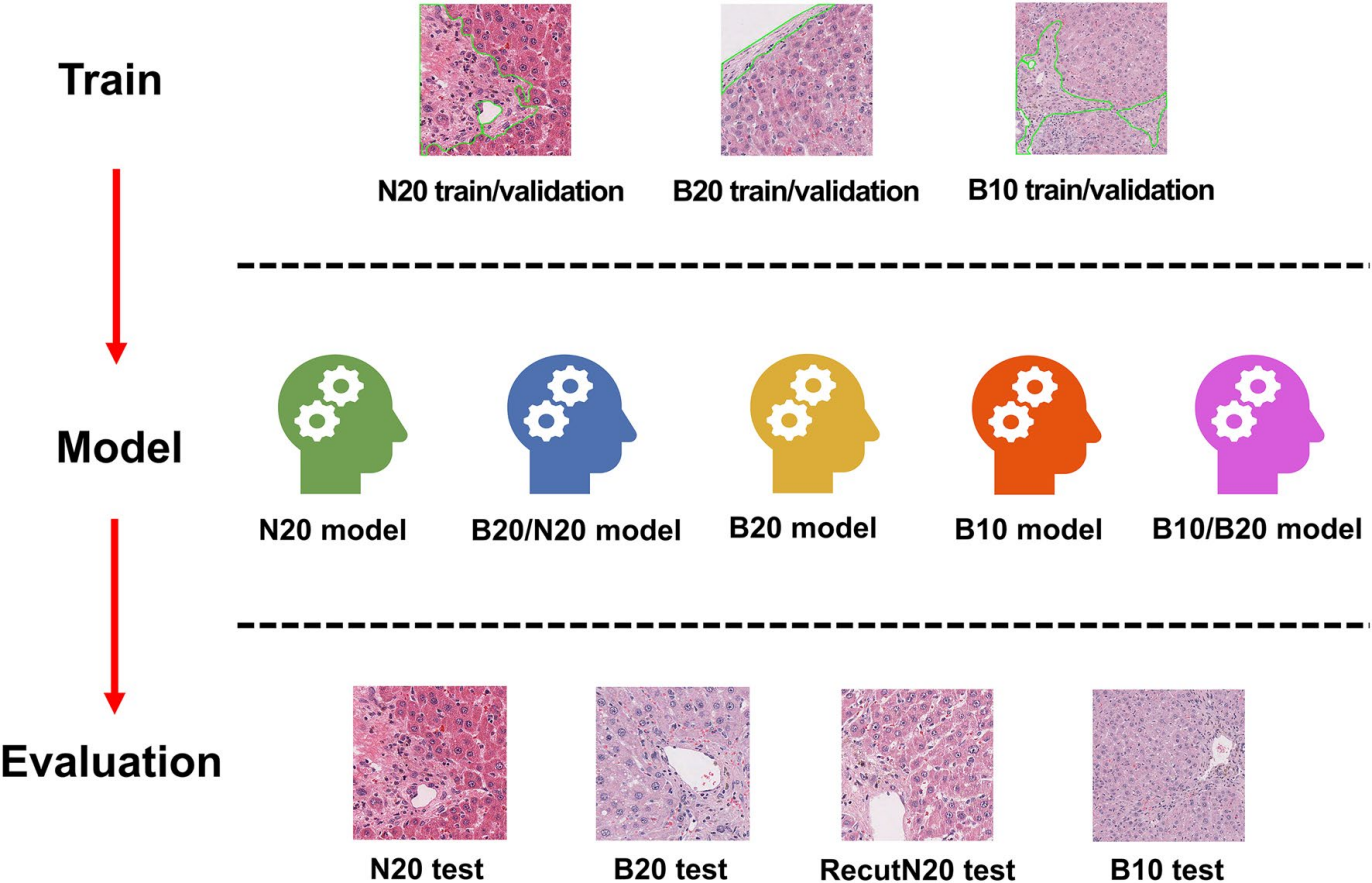
Scheme of the study procedure.

To investigate the effect of staining tones and magnification to predict pathological lesions in hepatic fibrosis, we trained the Mask R-CNN algorithm using three separate datasets, B10, B20, and N20, which vary in staining tones and magnification. We also trained the algorithm with a mixed dataset consisting of different staining tones (B20 and N20) or magnifications (B10 and B20). Five models were built according to this procedure and their performance in predicting hepatic fibrosis was evaluated using the test dataset for each dataset. In addition, to prove consistent model performance regardless of staining tone or magnification, we built two mixed models designated as the B20/N20 and B10/B20 models, which were trained with a mixed dataset of each model’s title. The performance of these two hybrid models was also evaluated using the test dataset and compared with other models (Fig. 1).

#### Metrics for model performance

**Figure 2:**
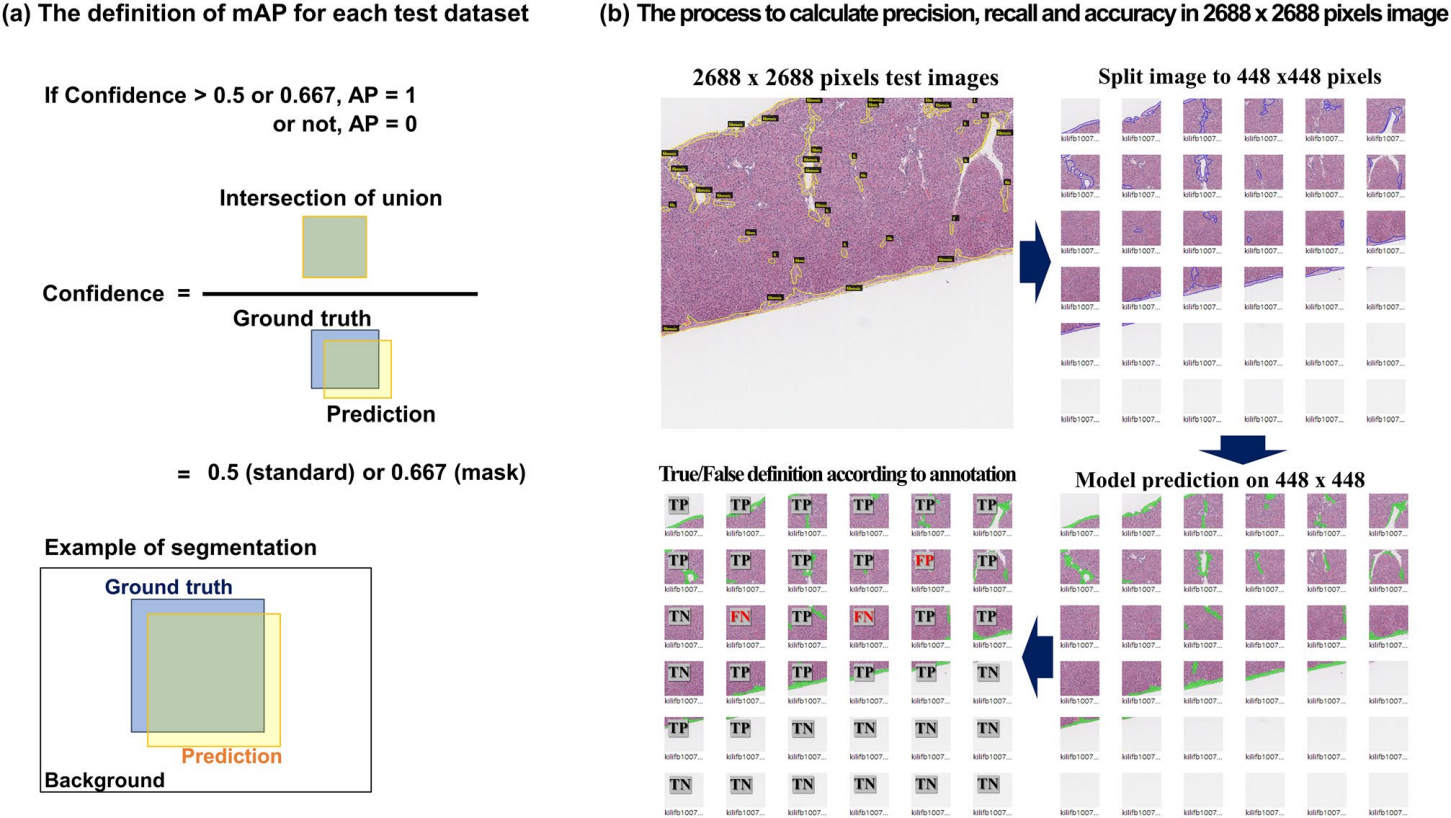
The definition of mAP used to assess the accuracy of trained models using test datasets (**a**). The process to calculate the precision, recall, and accuracy in order to evaluate each model’s performance in real-world data (**b**).

After model training, each model calculated the mean average precision (mAP) by comparing the ground truth annotation to the predicted lesion according to each model’s trained weight from the test dataset. Generally, mAP is defined according to precision and recall values; however, transformed mAP was used in this study^27^. This assumes an mAP value as 0 given any misprediction in an image based on a confidence of 0.5. Furthermore, we calculated the mask AP, which covers the mismatch of the number of predictions, in case the mask area is similar to the ground truth. In this case, the confidence was adjusted to 0.667, which is the value at which the intersection over the union between the prediction and ground truth is 80% (Fig. 2a).

In addition, B10, B20, and B10/B20 models were evaluated using 60 large-scale training images to represent their performance on the basis of real-world data. The 2688 × 2688 pixels of the images were divided into 448 × 448 pixels, and the trained model predicted the fibrosis lesions according to each trained weight. True and false were defined for each model according to the presence and absence of each predicted lesion, compared to the ground truth annotation (Fig. 2b). The precision, recall, and accuracy were calculated using Eqs. (1–3). Finally, to assess the accuracy of each predicted lesion, the number of pixels in the predicted area of a single or mixed dataset model and the ground truth annotation area were compared using linear regression, and each regression coefficient value, R^2^, was calculated.

1 $${\text{Precision}} = \frac{TP}{{TP + FP}}$$

2 $${\text{Recall}} = \frac{TP}{{TP + FN}}$$

3 $${\text{Accuracy}} = \frac{TP + TN}{{TP + FN + FP + TN}}$$

## Results

### Training results of test models

**Figure 3:**
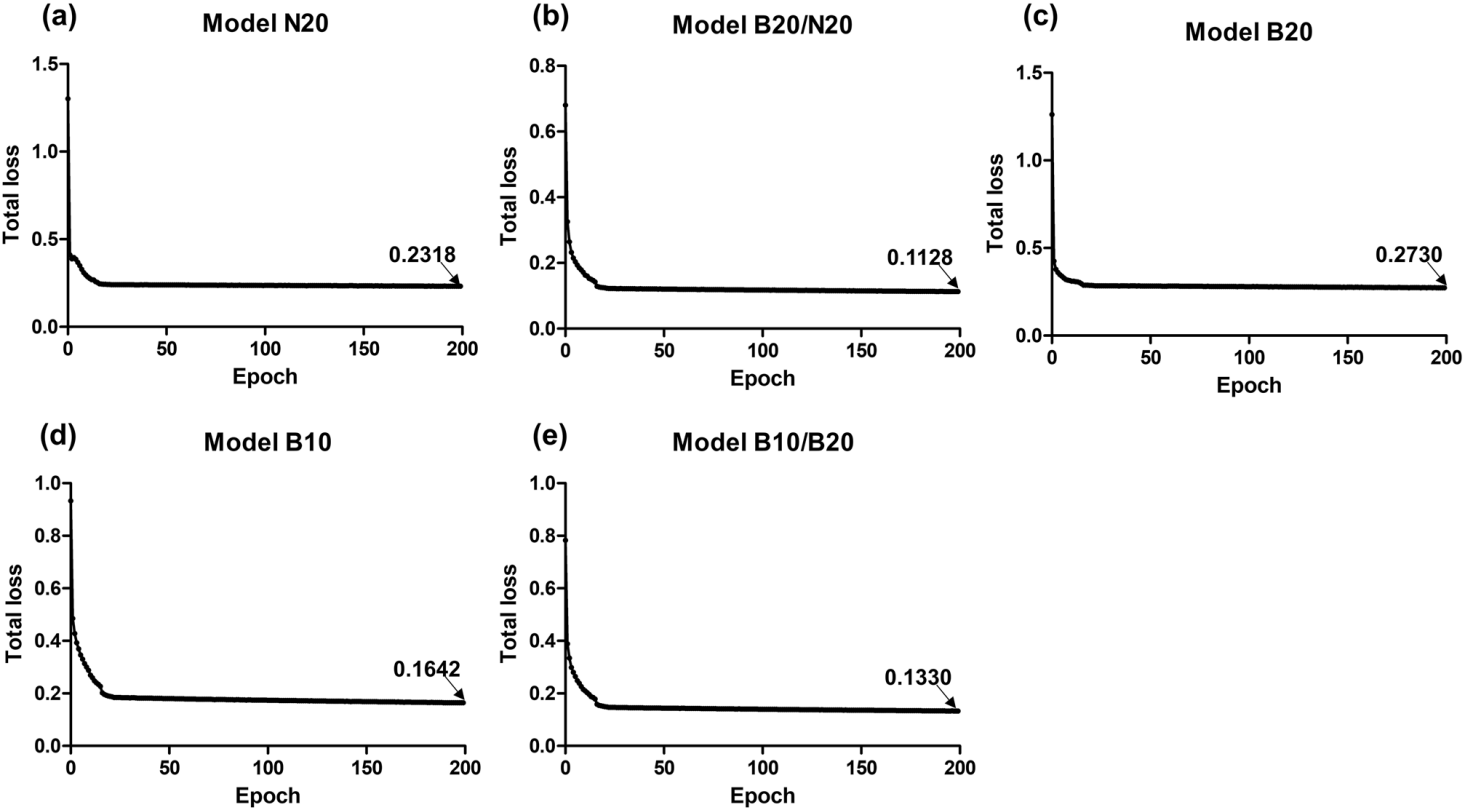
Total loss of each test model after the training.

Five test models were built, each trained with different dataset compositions and designated as models N20, B20, B10, B20/N20, and B10/B20, according to their trained dataset. Three of them (N20, B20, and B10) were trained with a single dataset with different staining tones or magnifications, and the other two models (B20/N20 and B10/B20) were trained with mixed datasets. Algorithm learning was successful as it showed stable loss values from the early phase to the end of the learning epoch. In particular, the mixed models B20//N20 and B10/B20 showed reduced loss at the end of the learning compared with the single dataset models (Fig. 3). The final loss value of model B20/N20 was 0.1128, which was 44.7% lower than the average final losses of models N20 and B20 (Fig. 3a–c). Model B10/B20 showed a 0.1330 final loss value, which was a 60.8% reduction from the average value of models B20 and B10 (Fig. 3c‒e). Therefore, the two models trained with mixed datasets of different staining tones or magnifications showed improved algorithm learning results than the models trained with a single dataset.

The numbers denoted with arrows in each graph are the final loss values at the end of training.

### Model performance according to staining tone

To verify whether the staining tones affected the model performance in predicting pathological lesions, we tested two single dataset models that differed in staining tones (N20 and B20) and the mixed dataset model (N20/B20). The test datasets included the dataset of each model and other datasets that differed in staining tone. In addition, we tested the RecutN20 test dataset, which had a similar staining tone to the B20 dataset, but the tissue itself was a serially sectioned slide of the N20 dataset. Testing RecutN20 could prove the consistent model performance of the N20/B20 model for predicting the pathological lesion to not only the different staining tones but also the variation in the study procedure.

As a result, the mixed dataset trained model (N20/B20 model), showed higher and stable mAP values than the single models that trained the N20 or B20 dataset only (Table 2). In contrast, single models showed good mAP values for their test dataset, poor values for another dataset, and neutral values for the RecutN20 dataset. The mask mAP value was also calculated, which can show the precision of the predicted region of the model regardless of the number of predicted lesions. We had a strict confidence value for determining whether the prediction was the correct answer. The mask mAP values of each model for the test datasets yielded higher values than the general mAP, except in some cases of single-dataset models. This might be due to the strict confidence parameter value of the mask mAP, which was adjusted from 0.5 to 0.667. These values reflect the relatively lower learning performance of the single dataset models than the mixed dataset model in Fig. 1.

**Table 2:** MAP values of test results according to the different datasets of each model.

	Model N20	Model B20	Model N20/B20
N20 test dataset	93.00 (96.00)%	56.0 (33.00)%	90.00 (98.00)%
B20 test dataset	61.00 (41.00)%	92.0 (97.00)%	91.00 (99.00)%
RecutN20 dataset	84.00 (73.00)%	84.0 (94.00)%	96.00 (97.20)%

The values in parentheses are masked mAP which calculated the accuracy of the mask region predicted by the model, compared to the ground truth annotations.

**Figure 4:**
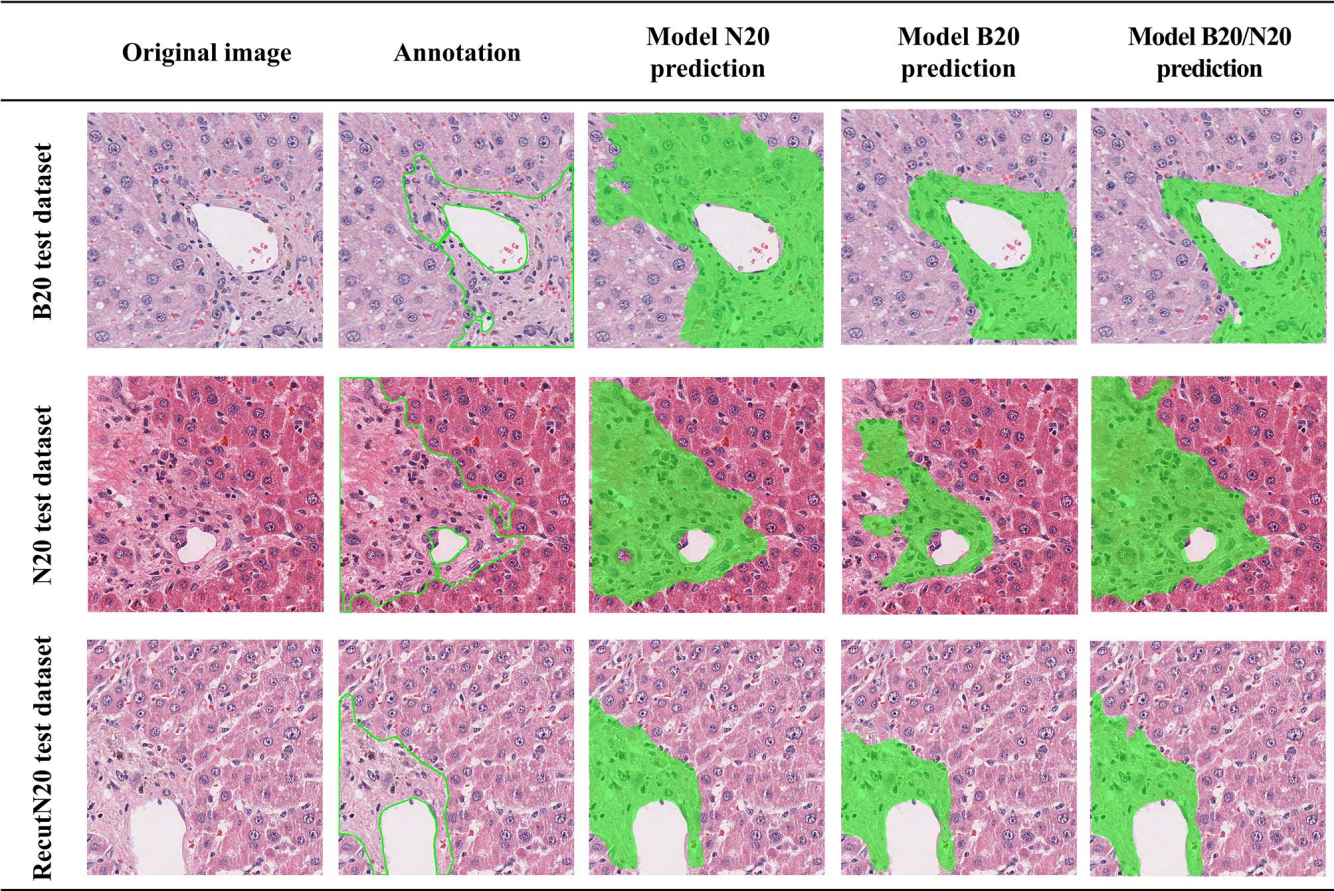
Prediction results of each model for the test dataset of its own and other datasets.

After calculating the mAP values, the actual prediction results for the test dataset images by each model were confirmed. The highest mAP values and learning results by the B20/N20 mixed dataset model consequently yielded optimal prediction results for hepatic fibrosis detection on the test dataset images than the single dataset models (Fig. 4). On the contrary, single models, especially model N20, showed worse prediction results for the test dataset images than any other model in the task of detecting hepatic fibrosis. This result is a consequence of the lowest mAP value for all test images, as shown in Table 2.

### Varying magnification affects model performance

#### Model performance according to slide magnification

To verify whether slide magnification affects model performance in predicting pathological lesions, we tested two single dataset models that differ in magnification (B10 and B20) and the mixed dataset model (B10/B20). The test datasets consisted of two datasets. The mixed magnification model yielded optimal mAP values for all datasets (Table 3). In particular, the mask mAP value of Model B10/B20 for the test dataset of B20 was 99.0%, which was almost similar to the ground truth annotation. Conversely, models trained using a single magnification dataset showed poor mAP values for the test dataset; in particular, the mAP values at low magnification were the lowest not only for the standard but also for the mask mAP.

**Table 3:** MAP values calculated from the test dataset of own or other datasets, in order to verify the effect of magnification of the image on model performance.

	Model B10	Model B20	Model B10/B20
B10 test dataset	76.00 (88.00)%	76.0 (64.00)%	85.00 (89.00)%
B20 test dataset	87.00 (94.00)%	92.0 (97.00)%	94.00 (99.00)%

The values in parentheses are masked mAP which represents the accuracy of the mask region as predicted by the model compared to the ground truth annotations.

**Figure 5:**
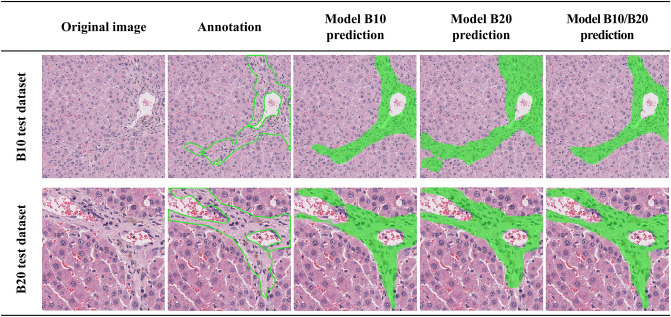
Model prediction results on the test dataset of its own and other datasets for the effect of staining tones.

In addition to the mAP values, the actual prediction results of the trained models were confirmed by comparing their predicted masks with the ground truth annotations (Fig. 5). The masks predicted the occurrence of hepatic fibrosis using trained models which showed consistent results with each mAP value.

### Mixed dataset trained model was more precise than single dataset trained model

**Figure 6:**
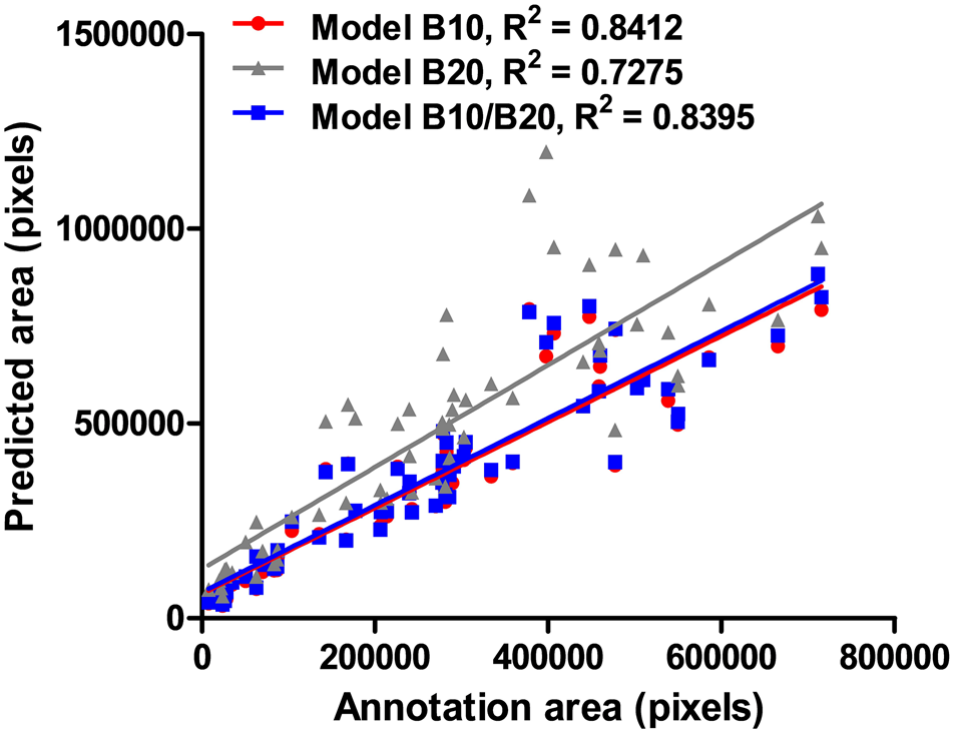
The correlation between the number of predicted hepatic fibrosis lesions and the ground truth annotation area.

**Figure 7:**
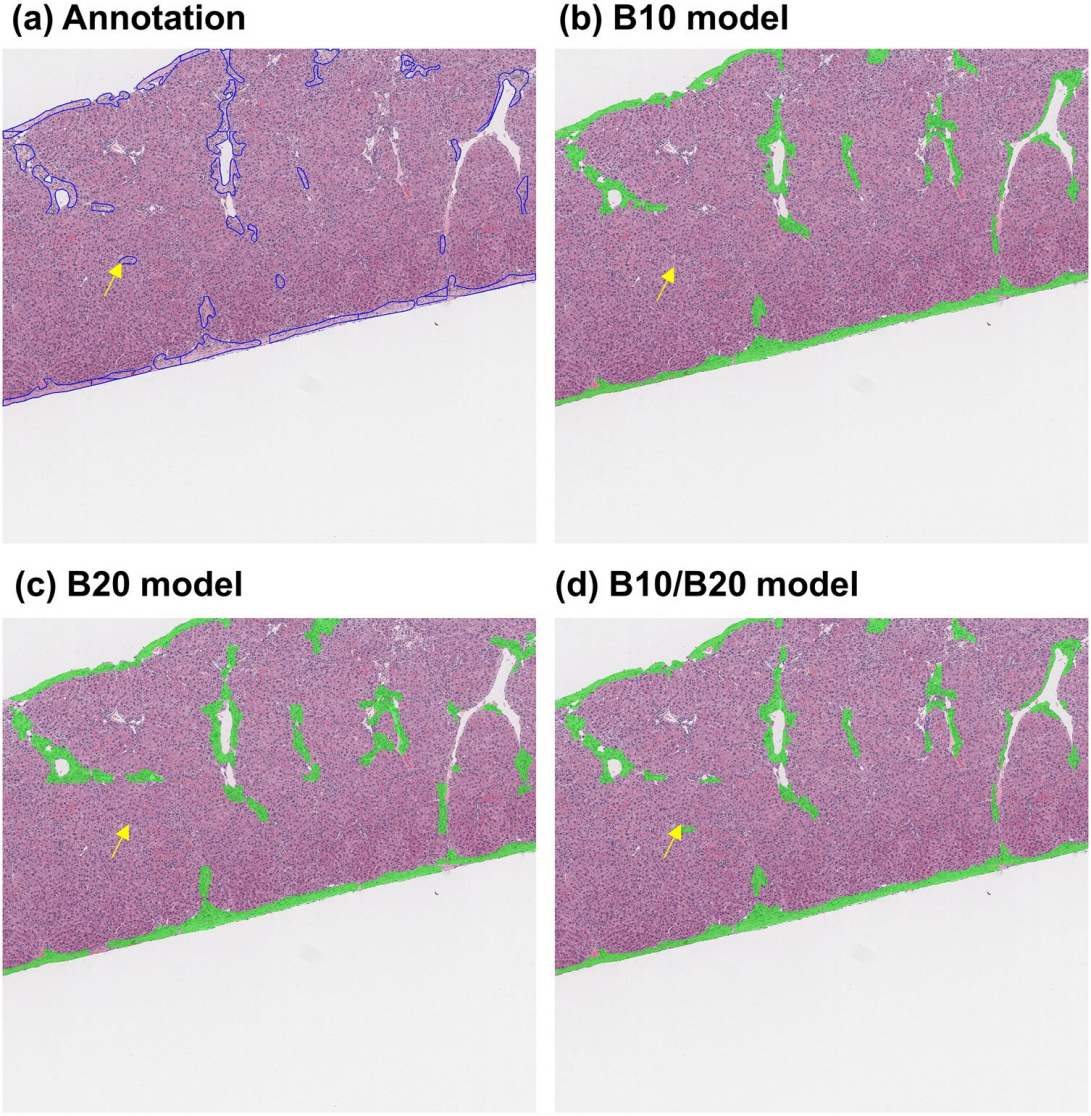
2688 × 2688 pixels images of the model prediction results, according to each trained weight. (**a**) Ground truth annotation, (b)–(d) predicted lesion by each single dataset model and the B10/B20 mixed model. The yellow arrows point out the fibrosis lesion that only B10/B20 model could detect.

To assess the performance of the mixed dataset trained model in a real-world context, we conducted an additional evaluation using sixty 2688 × 2688 pixels images (a quarter of the 10× scanned liver section). Precision, recall, and accuracy values were calculated according to the weights of each individual magnification-trained model and the mixed magnification-trained models. As a result, the B20 model showed the worst performance when compared to others, resulting in a lower accuracy in the test dataset. The performance of the B10/B20 model was comparable to the B10 model, in which the dataset had the same magnification as the test images (Table 4). This tendency has been reflected in the correlations between the number of pixels of fibrosis lesions predicted by models, and ground truth annotations (Fig. 6). The B10 and B10/B20 models showed high correlation to ground truth annotations with comparable R^2^ values, 0.8412, and 0.8395, while the value for the model B20 was 0.7275. These results differed from those of the mAP results, calculated from the test dataset, in which the mixed model showed superior accuracy. However, the B10/B20 model had an improved result compared to the B10 model. This tendency has been reflected in the actual prediction results on the 2688 × 2688 pixels image, as the B10/B20 model predicted hepatic fibrosis more precisely than others (yellow arrows in Fig. 7b). In summary, the mixed dataset models showed the most favorable prediction results, regardless of the magnification or staining tones for predicting hepatic fibrosis compared with ground truth annotation, and single-dataset-trained models still performed poorly.

**Table 4:** The accuracy, precision, and recall values of each model.

Parameters	Model B10	Model B20	Model B10/B20
Precision	0.7928	0.7883	0.7868
Recall	0.9512	0.8517	0.9611
Accuracy	0.8477	0.8069	0.8468

## Discussion

The implementation of AI algorithms to detect pathological lesions using H&E staining images from WSIs has been extensively discussed via the grand challenge of CAMELYON16, which was aimed at evaluating new and existing algorithms for the automated detection of metastases in lymph node sections^29^. The CAMELYON16 dataset consisted of images with or without nodal metastases verified by immunohistochemical staining provided by two different medical centers. The participants of the challenge should have considered the different magnifications and color variations for implementing an AI algorithm for the automated diagnosis of metastases. The leader boards of the challenge used multi-scale and different color blocks for the training stage of an algorithm and applied data augmentations to combat the rarity of tumor patches, in order to achieve optimal performance with reduced false negative rates for metastasis^7,30–32^. Studies on breast cancer and other previous studies considering the automated diagnosis of pathological image analysis have pointed out the importance of the variety of training image datasets for the improved performance of the AI model^33–37^.

In this study, the dataset's effect on the model's performance in predicting pathological lesions was investigated, using hepatic fibrosis as an example. The reduced total loss and high mAP of models trained by a mixed dataset of the different staining tones or magnifications showed a high concordance rate between the prediction masks and the ground truth annotations for detecting hepatic fibrosis. These results prove the importance of multiscale and staining color tones for the composition of a dataset for training the algorithm to predict the favorable detection result of the lesion of interest.

The quality of histopathological images can vary according to the procedure, from tissue fixation to slide scanning. Physically, it can be affected by tissue sectioning, fixation, type of fixative used, staining methods, chatter artifacts, and tissue folds on histopathological slides^38^. Furthermore, the quality of scanned images can vary due to errors in autofocusing, and variations in lightning and scanning conditions, leading to blurring, noise, and even differences, according to the vendors. Previous studies used stain normalization and color separation to overcome these differences in H&E-stained histopathological images and applied these techniques for training the artificial algorithm^39,40^. Our study proved that training the algorithm with different staining color tones showed improved performance in predicting hepatic fibrosis than single-color tone-trained models, without the color normalization. Even if single-color tone-trained models undergo augmentation, such as brightness modification, the variation in the original images might be more effective in training the AI model. In addition, model B20/N20 could consistently detect pathological lesions regardless of color tones from the detection test using the RecutN20 dataset, which serially sectioned the tissue of the N20 dataset and had a similar color tone to the B20 dataset.

For the test on the different magnifications of the models trained using additional magnification images, high-scaled images were better than lower-scaled images. This result is in concordance with our previous study^26^, in which the model trained using lower-scaled images (10×) showed improved performance when tested with high-scaled images (20×), and the results of other studies are also similar^41^. This lesion-specific characteristic has a threadlike shape, suggesting that segmenting the hepatic fibrosis in a high magnification environment could be more favorable than a low magnification. In addition, the mAP value of the model trained at 10× magnification in this study was lower than that in our previous study, owing to the lower number of images for the training dataset. Thus, this study also showed that the number of images in the dataset could affect the model performance.

The model performance of the mixed magnification dataset (10× and 20×) showed the most favorable prediction result with 99% of the mask mAP in detecting hepatic fibrosis. However, the test results from the large-scale images showed comparable performance between the B10 model, a single-dataset-trained model, and the B10/B20 model, while the B20 model showed poor performance in the test images, suggesting that this result might be the advantage of the same magnification between the B10 model and the large-scale test images. Nonetheless, the mixed-dataset-trained model (B10/B20 model) showed comparable performance with the B10 model in the prediction test with 2688 × 2688 pixels images. The B10/B20 showed a better value in the recall, which showed a better performance in segmenting the hepatic fibrosis fibers than the B10 model.

These results prove the importance of variations in staining color tones and multi-scales to improve the performance of the AI algorithm in the automated diagnosis of pathological lesions. Recent researchers proposed that the modified structures of the state-of-art AI algorithm is applied to the computer-aided diagnosis system using clinical imaging data, such as chest X-ray, for recognizing COVID-19^42,43^, cancer diagnosis from H&E images^44^, and grading glioma from MRI images^45^. The acquisition of imaging data from the clinical study is difficult, therefore modification or development of the algorithm is inevitable to get a good model performance. However, in the case of a non-clinical study, many slides are produced, allowing for the evaluation of the ratio of the lesion of interest between the vehicle and test group, in order to establish the toxicity of the test item. From this point of view, we suggest that the segmentation and quantification of the lesion of interest would be more important for the non-clinical study, and using the proper dataset for training rather than editing the algorithm architecture is more efficient in this study area. We concluded that training the algorithm with various staining color tones and multi-scaled image datasets would help make model performance more consistent in predicting pathological lesions of interest. In addition, we suggest the Mask R-CNN algorithm, an instance segmentation algorithm, which can be useful for quantification, as an appropriate algorithm that could well reflect these characteristics of the non-clinical study process. However, our research has a limitation that it cannot detect the multi-pathological lesions simultaneously in an image. Nevertheless, this study will provide reference for the elevation of model performance in the segmentation of pathological lesions for effective workflow in toxicological pathology.

## Supplementary Information

Supplementary Information.

## Data Availability

The datasets generated and/or analyzed during the current study are not publicly available due to its patent registration review but are available from the corresponding author on reasonable request.
