# Supplementary material for: Preparing pathological data to develop an artificial intelligence model in the nonclinical study
Source: Sci Rep. 2023 Mar 8;13:3896. doi: 10.1038/s41598-023-30944-x (PMC9994413; doi:10.1038/s41598-023-30944-x)

Preparing pathological data to develop an artificial intelligence model in the nonclinical study

Ji-Hee Hwang, Minyoung Lim, Gyeongjin Han, Heejin Park, Yong-Bum Kim, Jinseok Park, Sang-Yeop Jun, Jaeku Lee, and Jae-Woo Cho

Supplementary information

Figure legend

Supplementary Fig. S1. The comparison of RGB values between the B20 and N20 datasets from 50 sampled images of each dataset. All values showed statistical differences between the dataset ($p < 0.0001$); however, the value range between the dataset was obviously different for green and blue.

Supplementary Fig. S2. The representative images of studies B and N. The RGB values of each image had average values of the sample images.

Supplementary Fig. S3. The scheme of training Mask R-CNN algorithm. The training images with annotations went through the algorithm tuned by the hyperparameters modified as depicted in the scheme. As a result, the algorithm can segment the fibrosis lesion as the image on the right.

Supplementary Fig. S1

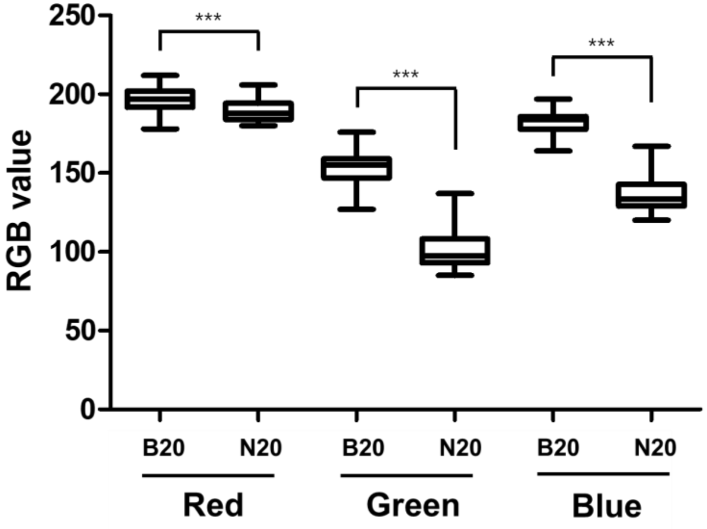

Supplementary Fig. S2

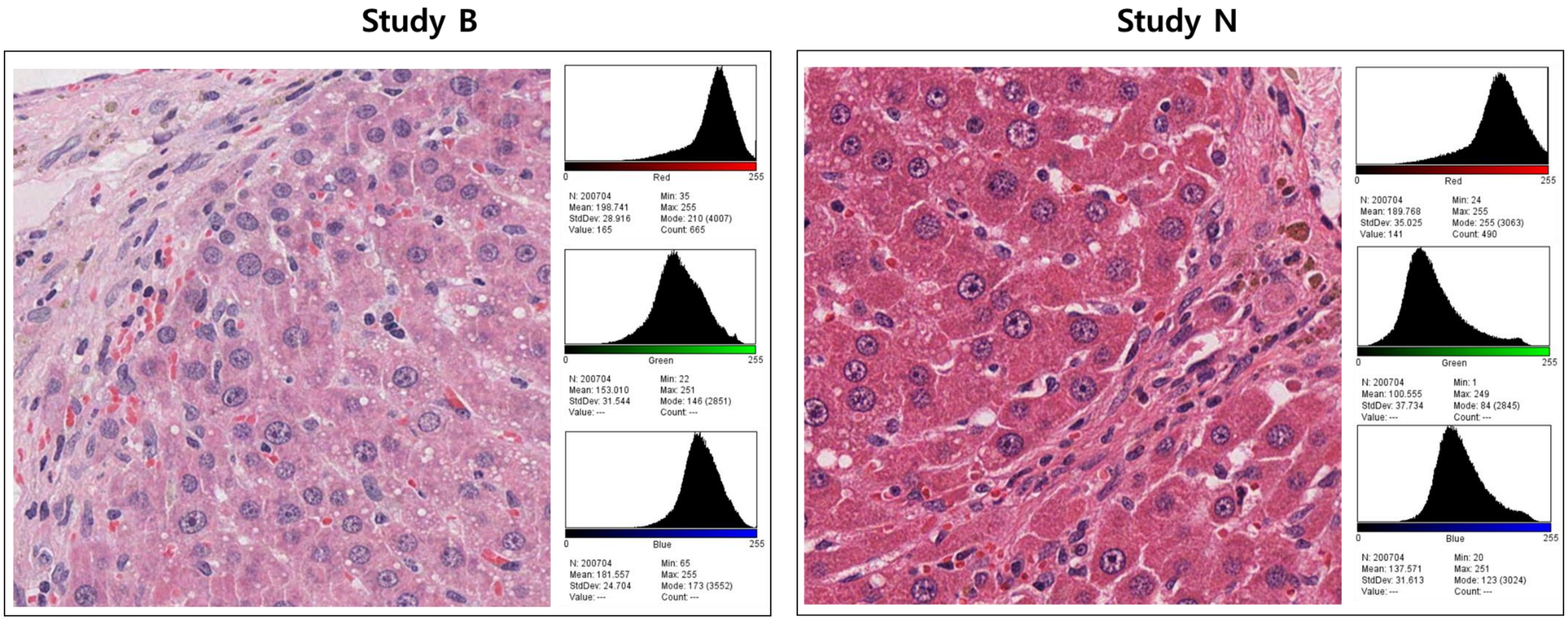

Supplementary Fig. S3

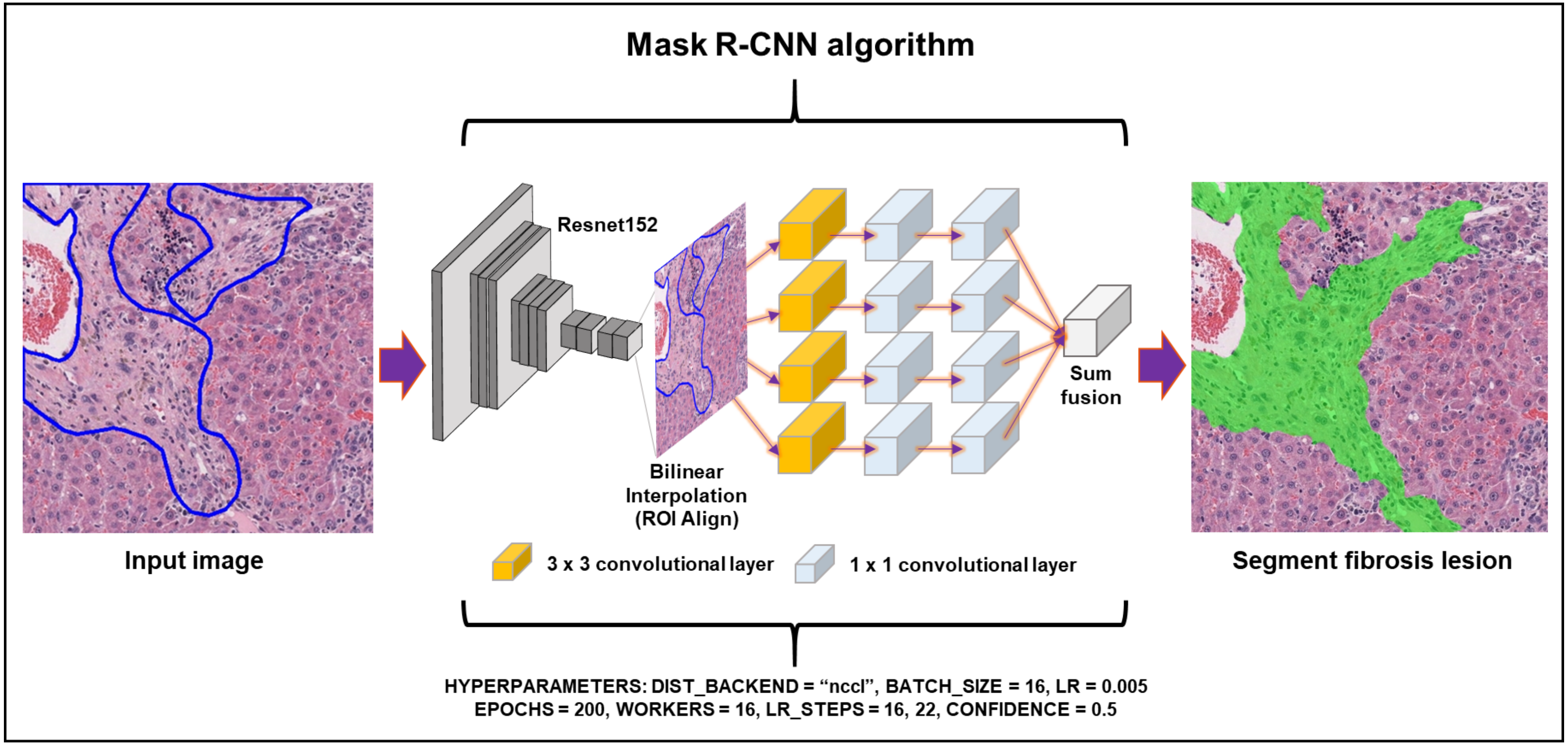

Supplement: Supplementary file 1 — Supplementary Information. [file 41598_2023_30944_MOESM1_ESM.pdf]
